# Case Report: Extreme Levels of Serum S-100B in a Patient with Chronic Subdural Hematoma

**DOI:** 10.3389/fneur.2012.00170

**Published:** 2012-12-05

**Authors:** Malin Elisabet Persson, Eric Peter Thelin, Bo-Michael Bellander

**Affiliations:** ^1^School of Medicine, The University of ManchesterManchester, UK; ^2^Department of Clinical Neuroscience, Section for Neurosurgery, Karolinska Institutet, Karolinska University HospitalSolna, Stockholm, Sweden

**Keywords:** S-100B, biomarkers, neurosurgery, human, malignant melanoma

## Abstract

The protein S-100B is a biomarker increasingly used within neurosurgery and neurointensive care. As a relatively sensitive, yet unspecific, indicator of CNS pathology, potential sources of error must be clearly understood when interpreting serum S-100B levels. This case report studied the course of a 46-year-old gentleman with a chronic subdural hemorrhage, serum S-100B levels of 22 μg/l, and a history of malignant melanoma. Both intra- and extra-cranial sources of S-100B are evaluated and imply an unclear contribution of several sources to the total serum concentration. Potential sources of error when interpreting serum concentrations of S-100B are discussed.

## Introduction

A 46-year old gentleman was admitted to hospital after having been found unconscious in his home. Past medical history included malignant melanoma that had been radically excised 9 years previously and the patient, after several controls, was believed to be in remission. There was no record of excessive alcohol- or other drug abuse.

**Figure 1:**
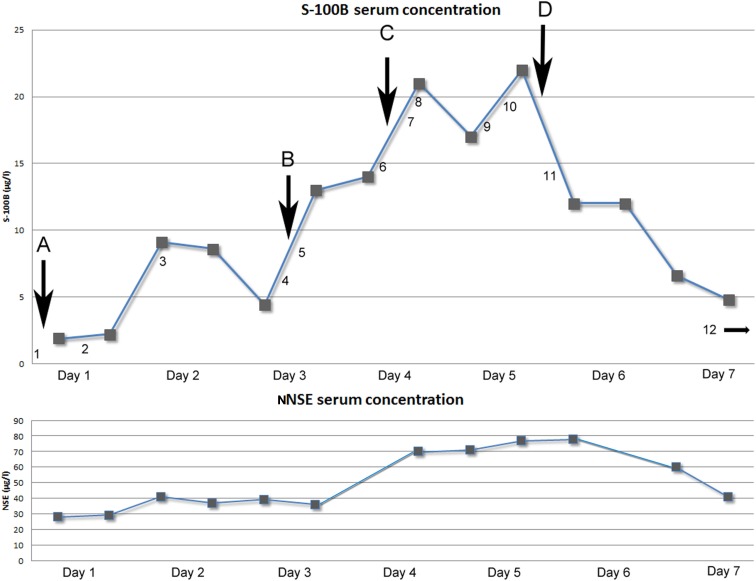
**Above: Graph showing serum concentration of S-100B (μg/l) over time (days)**. Arrows indicating neurosurgical interventions. (A) Evacuation of subdural mass (NCSP code AAD10). (B) Re-evacuation of subdural mass and placement of ICP-monitoring device (Codman^®^; AAD05, AAA20). (C) Insertion of external ventricular drain (AAF00). (D) Hemicraniectomy left side (AAK99). Numbers indicating performed radiological examinations [Computerized Tomography (CT) of the head if not otherwise stated]. (1) 15 mm midline shift to the right because of left side subdural mass. Old ischemic lesion in left side internal capsule (Figure 2). (2) Post-op evacuation, midline shift 12 mm, still compressed sulci. (3) Midline shift 11 mm, small frontal left-sided hematoma. Diffuse ischemic lesions left side occipital lobe. (4) Midline shift 15 mm, infarction in posterior cerebral artery left side and partially on the right side. (5) Post-op evacuation, middle shift 10 mm, unchanged ischemic lesions. (6) Midline shift 10 mm, better general conditions regarding intra-cranial mass effect, unchanged ischemic lesions. (7) CT-angiography: no signs of cerebral artery stenosis, dissection, or vasospasm. (8) Midline shift 10 mm, unchanged ischemic lesions. (9) CT-Thorax/Upper abdominal: 4.5 cm wide tumor in the lung, right upper lobe with growth to the mediastinum. Smaller cancerogenic lumps in the lung parenchyma. Multiple metastasis to the liver. (10) Magnetic resonance imaging (MRI): Midline shift 14 mm, compression of the brain stem. Infarctions in right side thalamus, internal capsule, pons, mesencephalon. Cortical infarctions left occipital lobe and right occipital lobe. Cerebellar infarctions (Figure 3). (11) Post-op hemicraniectomy. Thin line of blood beneath synthetic dura. Twelve millimeters midline shift. Unchanged ischemic areas. (12) (On day 8) Midline shift 16 mm, epidural hematoma with local mass effect. Brain stem herniation, compressed basal cisternae (Figure 4). Below: Graph showing the temporal profile of serum concentration of NSE (μg/l) over time (days). Two data points are missing due to hemolysis contaminating the sample.

**Figure 2:**
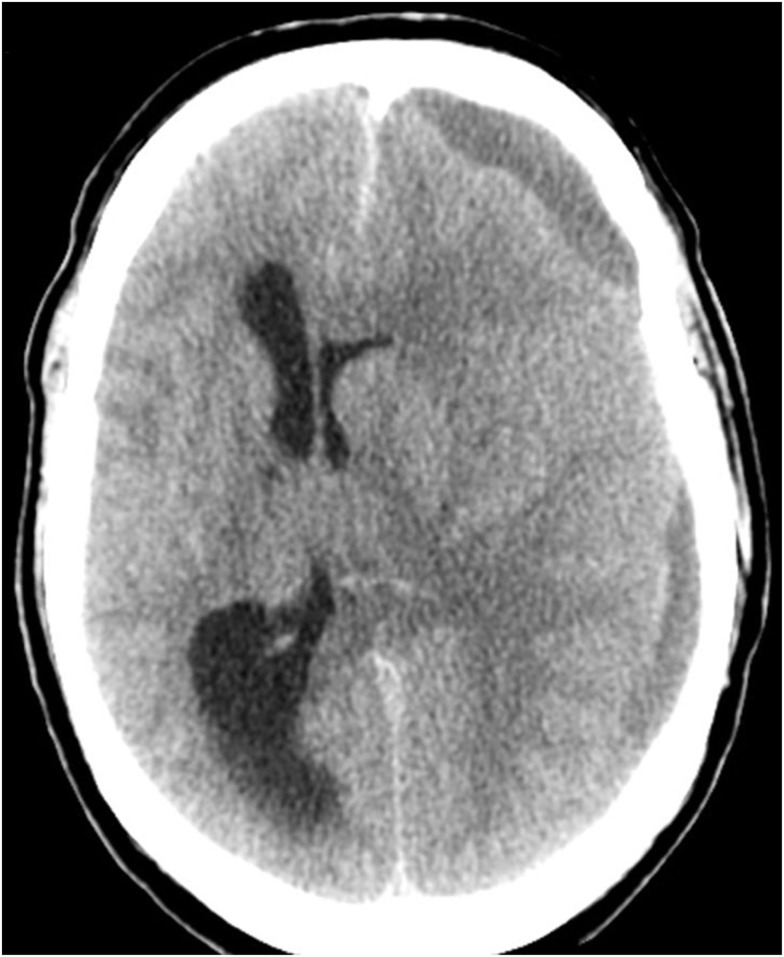
**Computerized tomography of the head (CT) on admission showing a left-sided sudural mass and a gross midline-shift of 15 mm**.

On admission the patient presented with a Glasgow Coma Score (GCS) of 3, a dilated left pupil, bilaterally brisk reflexes, and positive Babinski sign. There were no signs of neck stiffness, fever, petechiae, or trauma. S-100B was not acquired on admission (Figure 1). A CT scan showed a left-sided chronic subdural hemorrhage (SDH), resulting in a midline shift of 15 mm (Figure 2).

Evacuation of the subdural hematoma was performed. Intra-operatively, the pressure within the hematoma was noted to be low. The brain parenchyma failed to resume its natural position after the evacuation. The first S-100B sample was obtained post-operatively 4 h and 22 min after the paramedics were alerted. S-100B was elevated (1.9 μg/l; Figure 1). The patient did not respond as well as is expected after an SDH evacuation. A follow-up CT scan on day 2 revealed a persistent midline-shift, as well as new occipital infarcts, assumed to be secondary to cerebral herniation.

The patient improved to a GCS of 9 (E1 + M6 + V2; E, Eye; M, Motor response; V, Voice) on day 2. On day 4, the patient deteriorated, GCS 6 (E1 + M4 + V1). A CT scan revealed an increased midline-shift, so a re-evacuation was performed later the same day and an intra-cranial pressure (ICP) monitor (Codman^®^) was inserted. The dura was thicker than normal and the subdural mass did not look like a conventional hematoma, so samples were acquired for further pathological examination. Post-operatively the patient demonstrated a refractory critically unstable ICP ranging 15–30 mmHg and an increasing midline-shift on CT scan. Therapeutic management included hyperosmotic fluid, deep sedation with Midazolam, Morphine and Propofol, and hypothermia therapy. To facilitate treatment an external ventricular drain (EVD) was inserted. The S-100B levels were followed twice a day and showed an unusual peak of 22 μg/l (normal range <0.11 μg/l) on day 5 (Figure 1).

**Figure 3:**
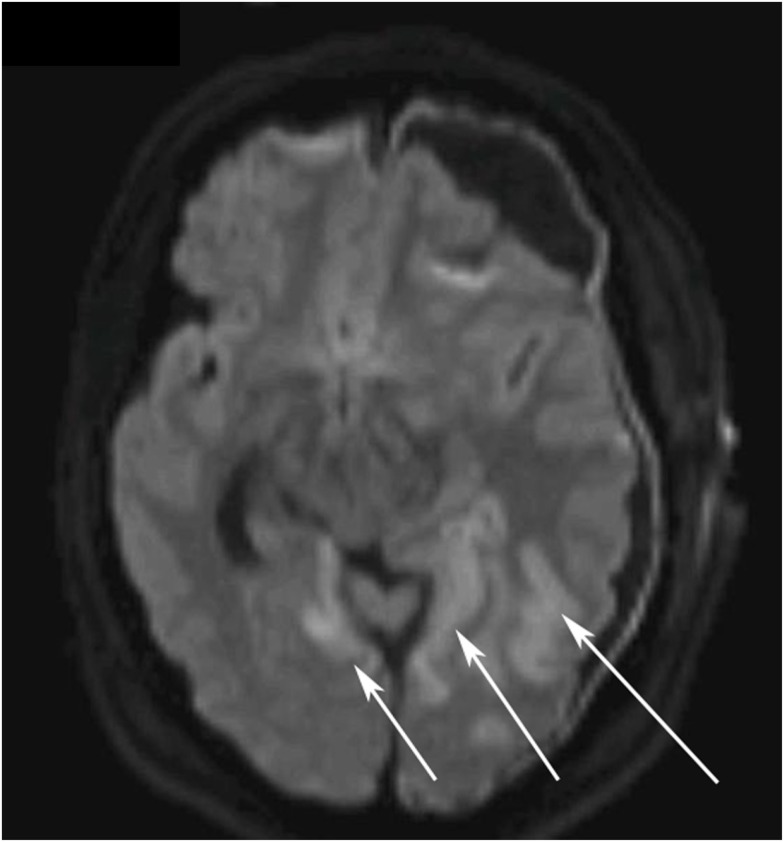
**Diffusion weighted magnetic resonance imaging (DWI MRI) of the brain on day 5**. The white arrows highlight the ischemic regions in the left and right occipital lobes.

Other sources of the extreme S-100B levels, including encephalitis, new cerebral infarctions, and extra-cerebral sources such as malignant melanoma, were pursued. Further head CT scans, including CT-angiography, showed no new findings. An EEG did not show any epileptic activity, MRI confirmed multiple infarcts, more than previously detected (Figure 3) yet no other information regarding the dura. A CT scan of thorax and abdomen showed widespread lung and liver metastases (not shown). Later day 5, a decompressive left-sided hemicraniectomy was performed (Figure 1, point E).

**Figure 4:**
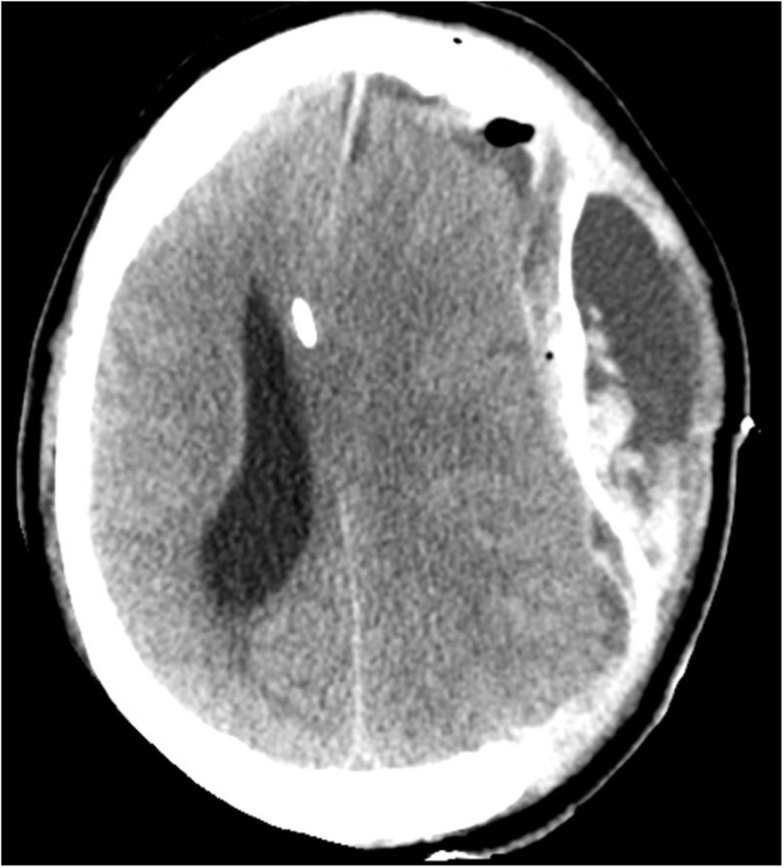
**CT on day 11, 4 days post a left-sided hemicraniectomy**. An EVD catheter *in situ* in the right hemisphere. There is a left-sided epidural expansive mass adjacent to the duraplasty (artificial membrane) causing a severe midline-shift.

While the S-100B declined post-operatively, the patient’s ICP was still refractory unstable and the midline-shift remained 15–16 mm (Figure 4). Despite optimized neurosurgical intensive care, the patient showed steadily increasing ICP so treatment was finally withdrawn. The patient passed away on day 8 of his hospital stay due to brain herniation.

The pathologist reported that extensive granulomatous tissue was noted within the hematoma and dura during the re-evacuation. The histopathological examination showed cells positive for S-100, Melan A, and HMB45, making metastases of malignant melanoma most probable. An autopsy confirmed malignant melanoma metastasis within the lungs, pleura, and lymphatic system. Metastatic tumor growth was also found in the brain parenchyma, dura, gallbladder, and kidneys.

## Background

The protein S-100B is an increasingly used biomarker within neurosurgery and neurointensive care. The calcium-binding protein, which is synthesized and released by cells mainly found in the CNS (Moore, 1982), is primarily an astrocytic protein but is also located in other cells of the CNS, such as neurons, oligodendrocytes, and choroid plexus epithelium (Steiner et al., 2007).

Both serum and CSF S-100B have been proposed to be valuable biomarkers of traumatic brain injury (TBI; Herrmann et al., 1999, 2001). In the early process, S-100B can help estimate the extent of the primary injury (Herrmann et al., 1999; Savola et al., 2004), monitor potential secondary insults (Raabe et al., 2004; Bellander et al., 2011) and be an asset in outcome prediction (Herrmann et al., 2001; Savola et al., 2004; Thelin et al., submitted).

Increased S-100B levels have also been found in patients suffering from ischemic cerebrovascular insults (Jonsson et al., 2001; Foerch et al., 2004). Vasospasm and long-term outcome in subarachnoid hemorrhages have been shown to correlate to increased levels of S-100B (Oertel et al., 2006). CNS infections may alter S-100B levels such as viral and bacterial meningoencephalitis and neuroborreliosis (Lins et al., 2005) and herpes simplex encephalitis (Studahl et al., 2009).

Clinically, S-100B is also the most widely used biomarker in malignant melanoma patients (Smit et al., 2008). The main use is monitoring of advance disease, staining of histopathological samples and to evaluate the efficacy of therapy (Martenson et al., 2001; Smit et al., 2008; Egberts et al., 2009).

Serum samples of S-100B were obtained on admission to Karolinska University Hospital and approximately every 12 h (06:00 and 18:00). All serum samples were arterial and analyzed using an automatic electro-chemiluminescence immunoassay (Elecsys S-100B^®^; Roche Diagnostics, Penzberg, Germany) method. The serum levels of S-100B and NSE, and radiological findings, were acquired using the medical files from the hospital database system Take Care^®^ (CompuGroup Medical Sweden AB, Farsta, Sweden). The ICP was acquired using ICU-Pilot^®^ (Dipylon Medical, Solna, Sweden).

## Discussion

This case report demonstrates a patient with remarkably high S-100B levels and complex CNS pathology of unknown origin. Throughout the NICU stay, frequent serum S-100B levels were sampled and used as an indicator of a potential underlying CNS injury and as a guide for management (Figure 1). There are, to our knowledge, no studies that investigate the relationship between chronic subdural hematoma and S-100B.

Regarding surgery and levels of S-100B, a study has shown that EVD insertion does not yield any significant increase of S-100B compared to base line levels (Woertgen et al., 2004) while hemicraniectomy has been shown to increase the levels of S-100B (Korfias et al., 2007). There appears to be a connection between increasing levels of S-100B in serum and the time of surgery B (re-evacuation) and C (insertion of EVD; Figure 1). It is difficult to determine if the first surgical procedure was a source of S-100B. as no sample was acquired prior to surgery. Nonetheless, there seem to be no obvious correlation between the hemicraniectomy (Figure 1, point D) and the 22 μg/l “peak” of S-100B. Other sources than the surgical trauma appears to influence the serum concentration.

S-100B has been shown to have a relatively short half-life, in studies between 30 and 90 min (Jonsson et al., 2000; Ghanem et al., 2001); hence, timing of S-100B sampling may be crucial. Worse outcome has been seen in TBI patients with increasing serum levels of S-100B (Pelinka et al., 2003) but this might be difficult to translate to the present case since no obvious traumatic intra-cranial lesions were present.

It is known that cerebral ischemia increases serum levels of S-100B. The development of several small cerebral infarcts is more likely to have contributed to the serum S-100B levels than potential traumatic lesions, in this case. CT-angiography performed during the period of high levels of S-100B (Figure 1, point 7) did not find any cerebral vasospasm that could contribute to increased serum concentrations. On day 2, the patient developed bilateral PCA infarctions. There is a possibility that there was a progress of the cerebral infarctions that was not seen prior to the performed MRI (Figure 3). In this patient cerebral ischemia and chronic SDH were the only detectable, as found by radiological examinations, source of S-100B from CNS.

Another factor supporting CNS as a source of S-100B in this patient is the Neuron Specific Enolase (NSE) levels that follow closely the trend of the S-100B concentration (Figure 1; *R*^2^ = 0.602, *p* < 0.001). NSE, a cytoplasmic glycolytic enzyme of neurons, generally follows the trend of S-100B after TBI and vascular cerebral injuries (Woertgen et al., 1997). In a study from 2003 there is shown that only raised serum levels of S-100B may indicate release from non-nervous tissues as a sign of potential multi-organ dysfunction, while NSE is more CNS specific (Kleine et al., 2003). Since NSE follows the trend of S-100B, it supports CNS as the main contributor of serum S-100B. A correlation has been found between increased ICP and the levels of S-100B and NSE (Olivecrona et al., 2009), but this did not yield serum concentrations of S-100B in the same magnitude as our patient’s. The ICP was elevated (above 20 mmHg) on several occasions, especially during day 5 to day 7.

S-100B has been suggested to be a marker of disturbed blood-brain barrier (BBB) integrity (Kapural et al., 2002), indicating that a damaged BBB may leak proteins, such as S-100B from the CSF into the blood and that this ratio may be used to quantify the BBB disruption. Other references point out that the serum levels of S-100B do not correlate to BBB integrity but instead probably reflect the true cellular damage, as seen in TBI (Bellander et al., 2011). The ratio between CSF-albumin and serum-albumin is commonly used to quantify BBB integrity (Tibbling et al., 1977). In this case the CSF levels of S-100B were low (4.2–13 μg/l) compared to what is seen after TBI (1649 ± 415 μg/l, mean ± SEM; Hayakata et al., 2004) and the ratio between CSF-serum-albumin were 0.001–0.002, clearly below 0.007 (Tibbling et al., 1977), the limit for disturbed BBB integrity. Hence, no BBB damage was indicated, making it less likely that the CNS is the only contributor to the serum concentration of S-100B in the present case.

Schwann cells, adipose tissue, muscle cells, myocardium, and melanocytes also contain S-100B (Anderson et al., 2001). S-100B is the most widely used biomarker in malignant melanoma patients where it is used to evaluate the efficacy of therapy (Martenson et al., 2001; Smit et al., 2008; Egberts et al., 2009). In serum concentrations, stage III malignant melanoma shows mean levels of 0.71 μg/l and the highest serum levels are seen in patients with visceral, bone, and/or brain metastasis (0.6–1.7 μg/l; Martenson et al., 2001). The present patient had a 3 cm × 2 cm wide and 0.9 mm thick malignant melanoma (Clarke grade III) on his shoulder that was radically excised 9 years previously. The follow-up controls did not show any new skin abnormalities and the patient was believed to be in remission. A CT scan did show widespread metastases in the liver and lung (not shown), confirmed by the clinical autopsy revealing a lymphatic spread and metastasis to the brain, dura, gallbladder, and kidney with a histological examination indicating malignant melanoma. The highest levels of S-100B were seen almost simultaneously as neurosurgical procedures were performed, processes that might have triggered a surge of S-100B into the serum affecting the total serum concentration.

Extremely high serum concentrations of S-100B have been reported preoperatively during cardiac surgery (40 μg/l; Anderson et al., 2001) and within an hour after severe TBI (22 μg/l; Thelin et al., submitted). One person with malignant melanoma showed a serum level of 126 μg/l, which was thought to be due to cell damage and necrosis of tumor sites (Ghanem et al., 2001), even S-100B levels as high as 221 μg/l have been reported in end stage melanoma patients (Martenson et al., 2001). Due to its short half-life, S-100B elevations seen in extra-cranial trauma are generally lower when samples are obtained later (median levels ranging 0.18–0.57 μg/l; Korfias et al., 2006; Stalnacke et al., 2006) than those seen in cerebral pathology such as TBI (mean levels ranging 1.7–4.01 μg/l; Herrmann et al., 2001; Savola et al., 2004) and ischemic cerebral infarcts (mean levels ranging 0.38–2.41 μg/l; Jonsson et al., 2001; Foerch et al., 2004) and extra-cranial S-100B generally do not contribute to the total concentration of S-100B if continuously sampled (Pham et al., 2010).

Our patient was exceptional with a late (day 5), steep increase (>20 μg/l) in serum levels of S-100B. Since S-100B follows NSE, some degree of CNS specificity is probable even though CSF levels of the biomarkers were low. There might have been amplification of the S-100B concentrations of intra-cranial origin due to the widespread malignant melanoma, especially from the tumor located within the dura where surgery might have induced S-100B release.

## Concluding Remarks

This case report of a gentleman with a chronic SDH, previous malignant melanoma, and sky-high S-100B levels serves as a reminder of potential extra-cerebral sources of error when utilizing S-100B as a biomarker.

## Conflict of Interest Statement

The authors declare that the research was conducted in the absence of any commercial or financial relationships that could be construed as a potential conflict of interest.
